# Effectiveness of an Advanced Naloxone Training, Simulation, and Assessment of Second-Year Pharmacy Students

**DOI:** 10.3390/pharmacy10060153

**Published:** 2022-11-19

**Authors:** Jennifer Courtney, Eugene Kreys, Bryan Luu, Tiffany Kreys, Ruth Vinall, Vy Quang, Erika Titus-Lay

**Affiliations:** College of Pharmacy, California Northstate University, 9700 W Taron Drive, Elk Grove, CA 95757, USA

**Keywords:** naloxone, pharmacy students, curriculum, protocol, overdose, opioids, team-based learning

## Abstract

*Background:* Opioid overdoses continue to be one of the most urgent public health priorities. In 2020, reported overdose deaths in the United States reached a high of over 93,000 cases. As the COVID-19 pandemic and opioid crisis continues to be addressed, life-saving agents must be more widely accessible to those with a high overdose risk. An essential step to increasing access is to train student pharmacists to dispense naloxone. Once licensed, the number of personnel authorized to dispense naloxone can increase. *Objectives:* To design a training program to educate second-year pharmacy (P2) students on furnishing naloxone under a state protocol. *Methods:* A multi-phased curriculum-based naloxone training program was delivered to P2 students and included lecture-based education, team-based learning (TBL) applications, case-based scenarios, and summative assessments to improve student knowledge and confidence in furnishing naloxone. Students were surveyed on their knowledge and confidence with naloxone prior to training, after the in-class training and TBL applications and after three assessments. Assessments included simulated patient counseling, case-based scenarios, and proper dispensing of naloxone in a community pharmacy simulation lab. *Results:* A total of 185 student pharmacists completed the naloxone training program and 68 completed all three surveys. Average scores for naloxone assessments were 83% for the APPS lab patient case, 90.5% for the prescription label typed for the naloxone product, and 88.5% for patient counseling. Statistically significant increases in knowledge-based quiz-like scores (42.1% after training vs. 7.2% after assessment) and in the proportion of students affirmatively answering survey questions after training and assessment was observed. *Conclusion:* Multi-phase curriculum-based naloxone training program improved pharmacy student knowledge and confidence in furnishing naloxone under a state BOP protocol.

## 1. Background

The COVID-19 pandemic has negatively impacted the overall health of Americans. As a result of the uncertainty and stress posed by the pandemic, as well as a disruption in treatment and recovery services, limited access to mental health services and support resources and deviations from standard day-to-day routines, a significant increase in prescription opioid use, overdose, and relapse has occurred, thereby elucidating the need for increased accessibility to life-saving treatments, such as naloxone [1,2]. On 13 March 2020, the COVID-19 pandemic was declared a national emergency with most states mandating a stay-at-home order [3]. The CDC reported a 30% increase in drug overdose deaths in the U.S. in the year 2020, with an all-time high of more than 93,000 cases, compared to the previous year [4]. As we continue to address the pandemic and the opioid crisis, we must prioritize making harm reduction agents more widely available to those with substance use disorders.

Since 1999, more than 932,000 Americans have died from drug overdoses and nearly 263,000 of those have lost their lives to prescription opioid overdoses [3,5]. Overdose deaths not only include prescription opioids, such as morphine, oxycodone, and hydrocodone but also include illegal opioids, such as heroin, and illegal drugs laced with synthetic substances, specifically fentanyl. Between 1999 and 2020, heroin-involved overdoses contributed to more than 13,000 deaths, which is a seven-fold increase in the number of heroin-involved overdose deaths in the U.S. [6]. In 2020, there were a reported 56,516 overdose deaths involving synthetic opioids, primarily fentanyl, which is a six-fold increase from 2015 to 2020 [7].

Pharmacists are the gatekeepers to much of the opioid availability in the U.S. and have been deemed the most accessible healthcare professionals, which has been quite beneficial during the COVID-19 pandemic. Each state has a protocol in place for pharmacists to dispense naloxone. This can include a state protocol, a statewide standing order by a state health officer, or a variety of other protocols, including collaborative practice agreements. Though U.S. laws and legislation have been making naloxone products readily available, opioid overdoses continue to be one of the most urgent public health priorities [8]. It is essential to emphasize the importance of naloxone as it is a critical tool to help reduce deaths involving opioid overdose [8]. Even though many states have been utilizing pharmacists to increase naloxone access, the number of opioid-involved overdose deaths remains high.

High rates of opioid-related deaths may be due to a limited access to naloxone in some communities. Although all 50 states have a protocol in place for pharmacists to dispense naloxone to patients, many pharmacists may be unaware of their state’s protocol or are untrained to provide naloxone to patients without a physician’s prescription. Additionally, opioid and naloxone use can be a sensitive and uncomfortable topic to talk about for both the individual and for the healthcare provider. Individuals may fear judgment from others and not obtain naloxone due to shame or embarrassment for using opioids. Naloxone stigma is considered a risk and major barrier for naloxone prescribing and access; thus, overdose prevention efforts should be aimed at reducing stigma to reduce negative perceptions [9,10]. A study by Rudolph et al. discussed additional barriers to dispensing naloxone in a community pharmacy setting, with the most commonly reported barrier being inadequate training and a need for further education on naloxone and opioid overdose prevention [11]. In addition, 95% of the surveyed pharmacists indicated that additional training would be helpful, including strategies for initiating discussions about naloxone and identifying eligible patients [11]. An essential step to increasing availability and access to naloxone is through training student pharmacists to dispense naloxone. Thus, upon licensure, an increase in the number of individuals authorized to dispense naloxone can be observed and more lives can be saved.

In 2015, California passed SB 493, which is legislation related to establishing pharmacists as healthcare providers. This piece of legislation authorizes trained pharmacists to furnish naloxone on their own authority [12]. The California Board of Pharmacy (BOP) requires pharmacists to complete at least 1 h of a naloxone continuing education program or an equivalent curriculum-based training program in a school of pharmacy before furnishing naloxone per state protocol [13]. While this legislation supports the integration of naloxone training into pharmacy curricula, this is not yet a widespread practice.

Several colleges of pharmacy have integrated naloxone training into their curricula and have demonstrated its effectiveness in improving student competency and confidence. Schartel et al. developed a naloxone training activity and assessed its impact on student pharmacist knowledge and confidence in counseling on opioid overdose management and naloxone administration [14]. For this study, first-year pharmacy students participated in a 50 minute naloxone training activity and a naloxone counseling case was incorporated into their Objective Structured Clinical Examination (OSCE), which was evaluated by a standardized patient using a knowledge and skills checklist and a global impression scale to assess the effectiveness of their communication. The 158 students who participated in the training agreed that OSCE training improved their confidence in counseling about the management of an opioid overdose and intranasal naloxone administration [14].

Musco et al. demonstrated that providing naloxone training can increase student confidence [15]. This group developed a teaching approach to naloxone education that incorporated active learning, technology, and interprofessional education components to train students on the proper administration of intranasal and intramuscular naloxone to a patient. For this study, third-year pharmacy students participated in a single 2 h class period followed by a 3 h skills laboratory that included a breathing emergency simulation activity the following week. Following completion of their training, student’s confidence in their ability to administer both naloxone formulations increased [15]. Bachyrycz et al. assessed the effectiveness of an in-class review of opioid overdose symptoms and risks as well as hands on practice with naloxone devices on student confidence in drug knowledge, patient counseling, and attitudes toward prescribing naloxone [16]. Student attitudes were evaluated using pre- and post-surveys administered to first- and third-year pharmacy students. Both groups demonstrated increased confidence levels in drug knowledge, clinical-type skills, and patient counseling following completion of the training [16].

Including naloxone training within the PharmD curriculum has also been shown to help improve interprofessional communication skills. In addition to the interprofessional education component incorporated by Musco et al., Kavanaugh et al. also included interprofessional education through the inclusion of academic detailing (AD), which allowed students to practice assessing patient and provider needs, respond to objections with non-biased and evidence-based information, practice applying a standing order, and enhance their understanding and demonstration of naloxone administration [17]. Simulating AD training, such as naloxone conversation starters, allowed the opportunity to practice handling hostility towards the use of naloxone to help combat physician resistance to naloxone dispensing [17].

Lastly, other studies have shown that providing naloxone training to students can improve knowledge and practice skills. Jacobson et al. compared the retention of overdose management knowledge and confidence in patient counseling between student pharmacists who only received a didactic lecture and those who received the same lecture plus a skills-based OSCE with a standardized patient [18]. Students were surveyed six months after the training and those who completed the OSCE did not demonstrate superior information retention nor confidence in counseling compared with students who only received a didactic lecture [18]. Donohoe et al. evaluated naloxone training provided to 130 pharmacy students that included a 1 h education session followed by a 3 h laboratory session that included mock counseling, case-based discussion, and opioid conversion scenarios [19]. Students completed pre- and post-surveys and the validated Opioid Overdose Attitudes Scale (OOAS). A majority of the 21 items on the OOAS statistically improved from baseline with the greatest improvements being the ability to inject naloxone and the effective management of an overdose [19].

While many studies have employed simulations and/or used case studies to promote student learning and confidence, not all aspects of naloxone training were included and, to our knowledge, no other study has used team-based learning (TBL) pedagogy to deliver the naloxone training. TBL pedagogy employs an active learning approach that places emphasis on self-directed learning, individual and team accountability, problem solving, and communication [20,21]. TBL is widely used in healthcare education today [22,23,24,25,26].

Collectively, the data supports the urgent need to increase naloxone training for pharmacists and embed naloxone training within the pharmacy curricula. The objective of the current study was to determine the effectiveness of implementing an expanded naloxone training program, which, unlike the studies described above, is an all-encompassing approach that covers a number of items, including: (1) identifying patients at risk of an opioid overdose, (2) choosing a patient-specific naloxone product from a case-based scenario, (3) reducing stigma, (4) providing patient counseling and education, (5) responding to an overdose, (6) learning to follow a statewide board of pharmacy protocol, and (7) using a community pharmacy simulation lab to replicate dispensing a prescription for naloxone.

## 2. Objective

The purpose of this study was to design and integrate a naloxone training program, in accordance with a board of pharmacy state protocol, into our pharmacy school curriculum. Using various teaching modalities, our aim was to determine pharmacy student practice readiness through the assessment of student knowledge, confidence, and ability to follow the state protocol for furnishing naloxone after participation in naloxone training, which included lecture-based education, TBL applications, case-based scenarios, and assessments during the second year (P2) of the pharmacy practicum curriculum. Naloxone training was provided during the P2 year of the pharmacy program to complement the didactic curriculum being delivered at that time, which includes topics related to pain management, with a focus on opioids, medication-assisted therapy, and harm reduction.

## 3. Methods

### 3.1. Educational Activity and Setting

Given the expanded scope of practice for pharmacists in California, a curriculum-based naloxone certificate training program was created to train student pharmacists to furnish and dispense naloxone. According to the protocol, students should be able to screen potential recipients, provide training in opioid overdose, properly select a patient-specific naloxone product, provide education and counseling on naloxone, offer resources or referrals for opioid addiction treatment to recipients, properly document the encounter and, if appropriate, dispense the product to the patient.

A naloxone training certificate program was provided to P2 students at California Northstate University College of Pharmacy during their fall semester practicum lab course. The naloxone certificate program was first developed and delivered in 2020 and repeated in 2021. Over that two-year period, 185 students were trained through this program. The professor involved in the development and implementation of this course was a community pharmacist with experience in dispensing naloxone to individuals in California and who also trained other pharmacists through California BOP training programs and other continuing education events. This certificate program was used to train student pharmacists to furnish naloxone to a patient, under a pharmacist’s supervision and without a physician’s prescription, under the California BOP protocol [27].

Prior to the training session a 28-question naloxone pre-training baseline knowledge and confidence survey was administered. Two survey questions asked about previous naloxone education or training, three survey questions captured demographic information, and the final twenty-three survey questions evaluated student knowledge and confidence in furnishing naloxone and the BOP state protocol. Of the 23 survey questions, 4 were knowledge-based quiz-like questions and 19 were 5-point Likert scale survey questions that were developed to assess self-confidence and familiarity with opioid overdose risks, patient-specific naloxone product selection, naloxone pharmacology, the BOP protocol, and comfortability of counseling and educating patients about opioid overdose and naloxone. The study lead, Dr. Jennifer Courtney, a practicing community pharmacist who is qualified to administer naloxone and who provides naloxone training to CNUCOP students, pharmacists, other healthcare professionals, and community stakeholders, developed the survey. The co-authors, 5 of whom are licensed pharmacists, reviewed the survey prior to administration to help ensure the questions were relevant.

**Figure 1:**
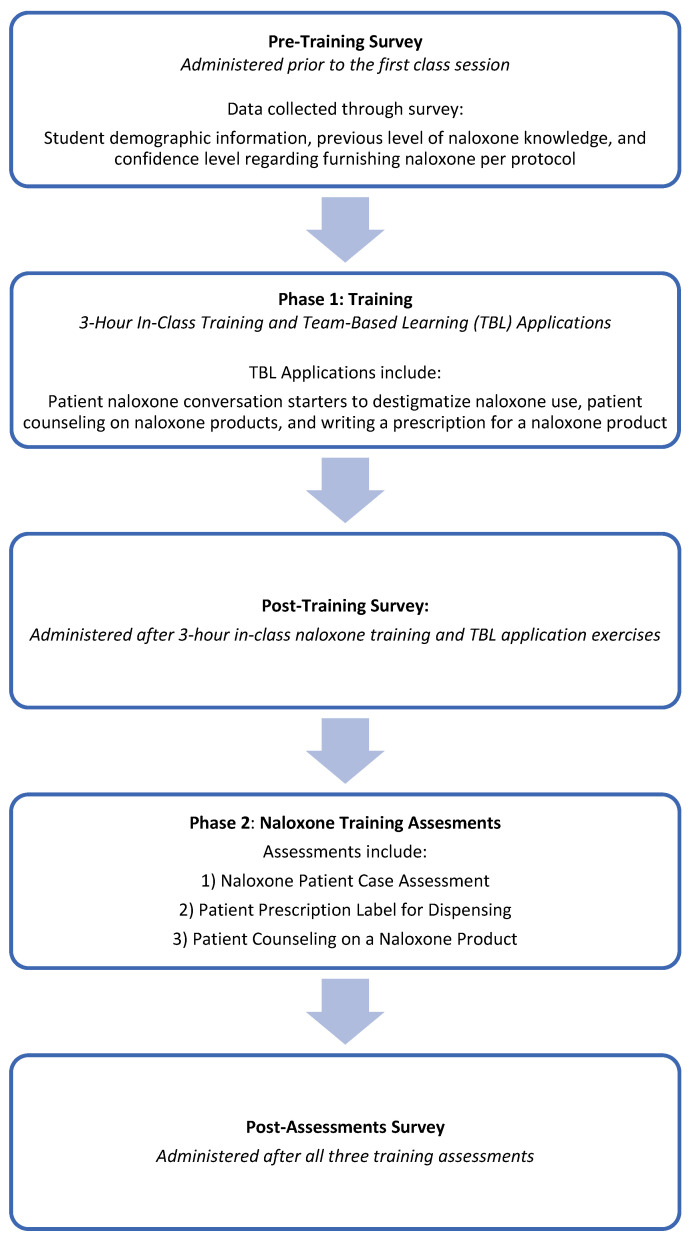
Naloxone training and survey sequence.

Three identical surveys were administered to students. The pre-training survey was given before the training session, the post-training survey was given after the 3 h in-class training session and TBL applications, and the final post-assessment survey was given after the three final assessments (Figure 1). More details on the assessments are provided below. Please note that the majority of CNUCOP classes are delivered using TBL pedagogy, meaning that students have already experienced TBL pedagogy in prior classes. No identifying information was collected and all surveys were completed anonymously via Survey Monkey. To link the three surveys, each student selected an unidentifiable self-chosen unique code. Informed consent for the study was obtained through a statement at the beginning of the survey and a checkbox stating, “I consent”.

The in-class naloxone training included a 1 h lecture-based educational session. After the 1 h naloxone training, a 2 h hands-on team-based application session was provided. Because naloxone stigma is considered a major barrier for naloxone prescribing, one of the team-based applications was designed to address the stigma surrounding naloxone access. Students completed a series of two team-based applications on (1) patient naloxone conversation starters and (2) patient counseling on naloxone products. In pre-established TBL teams, students were prompted to discuss ways to initiate conversations with patients about naloxone. Students were to discuss the benefits of their patient having naloxone on-hand at home and identify analogies or other ways to convey the importance of having naloxone without making patients feel stigmatized. Students were to choose non-stigmatizing language that promotes empathy and understanding and that reduces the perception of judgment. Students were then divided into groups of two to practice naloxone patient counseling. A third application was then given where students practiced documentation, including writing a new prescription for a naloxone product they were furnishing. A follow-up survey was given at the end of the 3 h class session and consisted of 23 of the 28, questions to determine their knowledge and confidence after the in-class training session. The 5 previous training and demographic questions were left out of the second and third surveys due to the ability to link the surveys by the student’s unique identifier chosen.

Participation in the in-class training, activity, and assessment was required as part of the practicum course; however, participation in the study was voluntary. This study was approved by the California Northstate University Institutional Review Board.

### 3.2. Evaluation and Assessment

Three summative assessments took place, including: (1) a naloxone patient case assessment, (2) the proper typing of a patient prescription label for dispensing, and (3) counseling of a patient on a naloxone product. The naloxone patient case assessment took place in our state-of-the-art Advanced Practice Pharmacy Simulation (APPS) Lab, which simulates a community pharmacy setting. The case was provided to students via a learning management system, CANVAS, and consisted of 6 questions that evaluated the student’s knowledge of opioid overdose risk factors, appropriate naloxone product selection based on patient-specific factors, procedures required by the BOP protocol, and specific drug information regarding naloxone. In addition, students had to properly type a prescription label for the naloxone product they selected for the patient, which was graded on choosing the correct patient, the most appropriate naloxone product for the patient, the proper number of doses required for furnishing under the BOP protocol, and the correct directions for the product. Naloxone patient counseling was scored by a faculty grader using a modified American Pharmacists Association (APhA) patient counseling rubric. The APhA rubric is used as a standard rubric by most pharmacy schools and for the APhA-ASP (Academy of Student Pharmacists) National Patient Counseling Competition. The rubric was modified for furnishing a naloxone product and consisted of a total of 46 points. Fourth-year pharmacy students served as standardized patients for the patient counseling.

### 3.3. Statistical Analysis

Baseline characteristics collected through the survey were presented as proportions. Knowledge-based quiz-like survey scores were presented as mean scores and standard deviations. Opinion-based Likert scale survey questions were presented as the proportion of subjects choosing to answer affirmatively, akin to “agreed” or “strongly agreed”, when referring to a statement. Normality of knowledge-based quiz-like survey scores was determined using the Shapiro–Wilk test. A comparison of knowledge-based quiz-like survey scores between two surveys administered before or after various phases of the course was conducted in a pairwise fashion using a paired t-test, while a comparison of opinion-based survey questions was conducted in a pairwise fashion using a Wilcoxon signed-rank test. An alpha level of 0.05 and a 95% confidence interval were selected to denote statistical significance. Cronbach’s alpha was calculated to assess the level of internal consistency for opinion-based surveys at all three phases of the course, with values ranging from 0 to 1, where 0 represented a complete lack of internal consistency and 1 represented perfect consistency.

## 4. Results

During the naloxone training in our 2020 and 2021 cohorts, a total of 107 subjects completed the study, with 107 completing the pre-training and post-training survey, only 68 completing the post-training and post-assessment survey, and 71 completing the pre-training and post-assessment survey. An examination of baseline characteristics revealed that about 15% of students underwent some form of previous naloxone training. If the student answered yes to having previous education or training on naloxone, a follow-up question was asked to describe the training. Most responses indicated informal training either through a self-study or through their Introductory to Pharmacy Practice Education (IPPE) community pharmacy rotation. Some responses indicated that they previously learned basic information about naloxone during interprofessional education (IPE) at another college and briefly during a pharmacotherapy course given at our university.

Demographic analysis of students surveyed found that about 63% of the subjects were female and 96% were younger than 35 years of age, with 66% self-identifying as Asian, 17% as Caucasian, 4% as Black, and 4% Hispanic. A statistically significant increase in the knowledge-based quiz-like scores was observed at every subsequent stage of the training, with the greatest increase of 42.1% observed after the in-class training phase of the course compared to a much smaller increase of 7.2% observed after the assessment phase of the course (Table 1). Likewise, a statistically significant increase in the proportion of students affirmatively answering survey questions was observed at nearly every subsequent stage of the course, with the largest increase observed after the training phase of the course compared to a much smaller increase after the assessment phase of the course. The only exception was for the question asking, “do you believe that utilizing pharmacists to increase naloxone access can save lives”, where a 3% reduction was observed in the proportion answering affirmatively on the post-assessment relative to the pre-training assessment, with no statistically significant difference observed (Table 2). Cronbach’s alpha was determined to be above 0.9 indicating very high internal consistency for the opinion-based surveys administered at all three phases of the training, thereby implying strong reliability of the surveys in general (Table 3).

Three summative assessments were given after the in-class training session. The average scores for the naloxone assessments were 83% for the APPS lab patient case scenario (lowest score = 2.3/6, highest score = 6/6), 90.5% for the typed prescription label for dispensing (lowest score = 4.5/10, highest score = 10/10), and 88.5% for the patient counseling (lowest score = 30/46, highest score = 46/46). The only difference between the two cohorts was that the in-class training and applications were virtually administered in 2020, due to the COVID-19 pandemic, and in 2021, they were given in person.

## 5. Discussion

The multi-phase curriculum-based naloxone certificate training program, which included lecture-based education, team-based learning applications, case-based scenarios, and summative assessments, was found to improve pharmacy student knowledge and confidence in furnishing naloxone under a state BOP protocol. Prior to the administration of the certificate training program, most students expressed discomfort towards naloxone, which may have resulted from an unfamiliarity with naloxone products. Once students were able to discuss the topic in class and further practice in the APPS lab, students demonstrated improved knowledge and comfort towards furnishing naloxone under protocol.

This study addresses important gaps in previous literature assessing naloxone education and training among pharmacy students. To our knowledge, this is the first study that evaluates such a robust naloxone training program and is also the first to utilize some TBL pedagogy in the training. The previously published six articles include some aspects of the individual training provided but none are all-encompassing like the training provided in our curriculum. We feel that the inclusion of an approach to addressing stigma, furnishing naloxone without a physician’s prescription, and the comprehensive, multi-phase training are the three key things that set our study apart. Schartel et al. assessed the patient counseling component but did not address the real-life simulation of preparing a naloxone prescription for dispensing to a patient. Kavanaugh et al. assessed the effectiveness of naloxone education and preparing students to dispense via a standing order through use of academic detailing. Though it is crucial to learn this skill of providing evidence-based education to physicians in order to improve the quality of care and patient outcomes, a physician’s prescription for naloxone is not generally needed in most states that now operate under a standing prescription order or pharmacist furnishing protocol. Because of this, academic detailing may not be a necessary skill for the dispensing of naloxone. The study by Musco et al. focused on the training of pharmacy students to be able to administer naloxone but did not train students on appropriate furnishing under a board of pharmacy protocol. Given that community pharmacy positions are projected to remain at about 60% of the overall pharmacy workforce through at least 2026, it is equally important to train our student pharmacists who will be filling these community pharmacy roles to have a greater impact on naloxone access [28]. The naloxone training provided by Bachyrycz et al., Jacobson et al., and Donohoe et al. is most similar to the training provided in our college of pharmacy. The training described in the studies by Bachyrycz et al. and Jacobson et al. did not appear to include the simulation of dispensing nor team application-based learning. Donohoe et al. reported the inclusion of a case-based discussion but also did not appear to include a simulation of dispensing. In addition to the differences described above, the previous articles also do not address the stigma associated with naloxone dispensing. Though Kavanaugh et al. incorporated simulated activities based on real-life scenarios that pharmacists may encounter, the simulations included academic detailing to respond to provider barriers and did not include scenarios where patient barriers required identification and consultation. Bachyrycz et al. assessed student attitudes toward prescribing of naloxone but this does not directly translate to stigma. Furthermore, our study is the only one that discusses the use of simulated prescription entry on a pharmacy software for students to practice the documentation and prescription processing piece of naloxone furnishing. Our study was also one of the two that assessed student knowledge and comfort, specifically with furnishing naloxone, in addition to the study by Kavanaugh et al.

Our opinion-based surveys had excellent internal consistency as demonstrated by the Cronbach’s alpha score. Based on our data, it was determined that students found most benefit from the in-class lecture and team application-based scenarios. Of the students surveyed after the completion of the in-class naloxone education training and the TBL application session, a significant improvement in confidence, knowledge, and comfort in educating and counseling patients on naloxone was observed. The summative assessments were found to provide further improvement in student-reported knowledge and comfort with naloxone furnishing, though to a lesser extent than the in-class lecture and team application-based scenarios. Though students’ perceived benefit from the summative assessments were not as strong, it allowed for a structured and objective assessment of students’ skill and knowledge in following a BOP protocol on furnishing naloxone and also on providing patient counseling. In addition, the modified APhA patient counseling rubric used to score the students could easily be implemented by other colleges of pharmacy to objectively grade student performance on naloxone counseling.

Limitations that were identified include the small total number of participants (*n* = 68) that completed all three surveys and the subjective nature of Likert questions that might allow for students to have an unrealistic understanding of their knowledge or comfort with furnishing naloxone. Survey fatigue may have contributed to the lower number of students completing all three surveys, since two were completed on the same day and the last completed just one week later. Though awareness of stigma was addressed through one of the application-based cases and through patient counseling, it was not assessed within the survey questions. Moreover, the case-based scenario summative assessment included only six points and did not include questions on all of the available naloxone products. For future offerings of the training, we plan to improve these limitations through the inclusion of more questions on the survey that specifically assess stigma. Though the patient case assessment was comprehensive, we plan to make further enhancements to increase the number of points available for the assessment and allocate points based on the difficulty of the question. We also plan to include questions for all of the available naloxone products to better assess knowledge and comfort with each formulation available. In addition, we plan to require remediation for all students that do not pass the summative assessments. Though the average grades for each of the three summative assessments was a grade of B or higher, there were students that did not successfully pass at least one of the three assessments. Because the syllabus for the course did not outline a remediation plan, we were unable to require remediation for these.

## 6. Conclusions

This multi-phase curriculum-based naloxone training program improved pharmacy student knowledge and confidence in furnishing naloxone under a BOP protocol. Pharmacy students undergoing such training may be better situated to furnish and counsel on naloxone and opioid overdose prevention upon graduation as a result of this enhanced naloxone training program, thus reducing barriers to obtaining naloxone. While the study demonstrated the effectiveness of the naloxone training program, the survey results have a limited generalizability as a result of low study participation and because the study was conducted at a single university. The results should be validated at other universities with multiple cohorts. Assessments should be expanded to include the subsequent second-year pharmacy cohort and their responses and stigma should also be assessed in the survey questions. Finally, given that individual states have their own varying protocols for standing orders or pharmacist furnishing of naloxone, the training provided in this certification program may not be able to be applied across all colleges of pharmacy. Although, the training’s transferability is high and could be modified to fit other state protocols, as the naloxone knowledge is the same.

## Figures and Tables

**Table 1:** Increase in knowledge-based survey scores.

N	Phase of Course	Mean Score ± s.d. (%)	Percent Change (95% CI)	*p*-Value
107	Post-training	73.4 ± 2.6%	+42.1% (35.6–48.8%)	<0.001
	Pre-training	31.2 ± 3.0%		
68	Post-assessment	80.6 ± 2.2%	+7.2% (2.8–11.7%)	0.002
	Post-training	73.4 ± 3.0%		
71	Post-assessment	80.0 ± 2.5%	+51.5% (43.5–59.4%)	<0.001
	Pre-training	28.2 ± 3.9%		

**Table 2:** Proportion that affirmatively answered opinion-based survey questions.

	Change in the Proportion Answering Affirmatively * between Post-Training and Pre-Training (*p*-Value)	Change in the Proportion Answering Affirmatively * between Post-Assessment and Post-Training (*p*-Value)	Change in the Proportion Answering Affirmatively * between Post-Assessment and Pre-Training (*p*-Value)
How familiar are you with the risks of opioid overdose? ^a^	54% (<0.001)	7% (<0.001)	61% (<0.001)
How familiar are you with the pharmacology of naloxone? ^a^	69% (<0.001)	10% (<0.001)	78% (<0.001)
Do you believe that utilizing pharmacists to increase naloxone access can save lives? ^b^	6% (<0.001)	−3% (0.493)	3% (0.005)
How familiar are you with the CA BOP protocol for pharmacists to furnish naloxone? ^a^	81% (<0.001)	6% (0.004)	87% (<0.001)
How comfortable do you feel talking to patients about opioids? ^c^	61% (<0.001)	11% (<0.001)	72% (<0.001)
How comfortable do you feel talking to a patient about naloxone? ^c^	60% (<0.001)	11% (<0.001)	71% (<0.001)
How comfortable do you feel counseling a patient on the administration of intramuscular naloxone in a vial with a syringe? ^c^	57% (<0.001)	12% (<0.001)	69% (<0.001)
how comfortable do you feel counseling a patient on the administration of commercially available Narcan nasal spray? ^c^	67% (<0.001)	8% (<0.001)	75% (<0.001)
How comfortable do you feel counseling a patient on the administration of naloxone nasal spray kit with atomizer/white cones? ^c^	62% (<0.001)	7% (<0.001)	70% (<0.001)
How comfortable do you feel counseling a patient on the administration of Evzio? ^c^	69% (<0.001)	12% (<0.001)	80% (<0.001)
How comfortable do you feel selecting the proper naloxone product and route for a patient? ^c^	73% (<0.001)	10% (<0.001)	83% (<0.001)
How comfortable do you feel counseling a patient on the effectiveness of naloxone? ^c^	71% (<0.001)	7% (<0.001)	78% (<0.001)
How comfortable do you feel counseling a patient on the adverse effects of naloxone? ^c^	72% (<0.001)	6% (<0.001)	78% (<0.001)
How comfortable do you feel counseling a patient on safety and tolerability of naloxone? ^c^	73% (<0.001)	8% (<0.001)	81% (<0.001)
How comfortable are you with counseling a patient on opioid overdose prevention? ^c^	66% (<0.001)	9% (<0.001)	75% (<0.001)
How familiar are you with identifying the signs and symptoms of opioid withdrawal? ^a^	75% (<0.001)	7% (<0.001)	83% (<0.001)
How familiar are you with identifying the signs and symptoms of an opioid overdose? ^a^	78% (<0.001)	6% (<0.001)	84% (<0.001)
How comfortable would you feel responding to an opioid overdose? ^c^	77% (<0.001)	6% (<0.001)	83% (<0.001)
How comfortable do you feel writing a prescription for naloxone? ^c^	81% (<0.001)	6% (<0.001)	86% (<0.001)
How comfortable do you feel typing a new prescription for naloxone? ^c^	67% (<0.001)	4% (<0.001)	71% (<0.001)

^a^ Answer options: not at all familiar, slightly familiar, somewhat familiar, * moderately familiar, * extremely familiar. ^b^ Answer options: strongly disagree, disagree, neither agree or disagree, * agree, * strongly agree. ^c^ Answer options: not at all comfortable, slightly comfortable, somewhat comfortable, * moderately comfortable, * extremely comfortable. Please see Supplemental Tables S1–S3.

**Table 3:** Cronbach’s alpha reliability test for opinion-based surveys.

	Pre-Training	Post-Training	Post-Assessment
Cronbach’s alpha	0.952	0.970	0.973

## Data Availability

The data presented in this study are available on request from the corresponding author.

## Supplementary Materials

The following supporting information can be downloaded at: https://www.mdpi.com/article/10.3390/pharmacy10060153/s1, Table S1: Survey results comparing student perception pre-teaching and post-teaching, Table S2: Survey results comparing student perception post-teaching and post-assessment, and Table S3: Survey results comparing student perception pre-teaching and post-assessment.
