# Supplementary material for: Effectiveness of an Advanced Naloxone Training, Simulation, and Assessment of Second-Year Pharmacy Students
Source: Pharmacy (Basel). 2022 Nov 19;10(6):153. doi: 10.3390/pharmacy10060153 (PMC9680519; doi:10.3390/pharmacy10060153)
Supplement: Supplementary file 1 [file pharmacy-10-00153-s001.zip › pharmacy-1988005-supplementary.pdf]

Table **S1.** Survey results comparing student perception pre-teaching and post-teaching.

	**Pre-teaching answering in the affirmative**	**Post-teaching answering in the affirmative**	**Change in the proportion answering in the affirmative post-teaching and pre-teaching (p-value)**
How familiar are you with the risks of opioid overdose?	36%	90%	54% (<0.001)
How familiar are you with the pharmacology of naloxone?	17%	86%	69% (<0.001)
Do you believe that utilizing pharmacists to increase naloxone access can save lives?	92%	98%	6% (<0.001)
How familiar are you with the CA BOP protocol for pharmacists to furnish naloxone?	7%	88%	81% (<0.001)
How comfortable do you feel talking to patients about opioids?	24%	86%	61% (<0.001)
How comfortable do you feel talking to a patient about naloxone?	27%	86%	60% (<0.001)
How comfortable do you feel counseling a patient on the administration of intramuscular naloxone in a vial with a syringe?	15%	72%	57% (<0.001)
how comfortable do you feel counseling a patient on the administration of commercially available Narcan nasal spray?	23%	89%	67% (<0.001)
How comfortable do you feel counseling a patient on the administration of naloxone nasal spray kit with atomizer/white cones?	17%	79%	62% (<0.001)
How comfortable do you feel counseling a patient on the administration of Evzio?	9%	77%	69% (<0.001)
How comfortable do you feel selecting the proper naloxone product and route for a patient?	10%	83%	73% (<0.001)
How comfortable do you feel counseling a patient on the effectiveness of naloxone?	19%	90%	71% (<0.001)
How comfortable do you feel counseling a patient on the adverse effects of naloxone?	19%	90%	72% (<0.001)
How comfortable do you feel counseling a patient on safety and tolerability of naloxone?	15%	88%	73% (<0.001)
How comfortable are you with counseling a patient on opioid overdose prevention?	21%	87%	66% (<0.001)
How familiar are you with identifying the signs and symptoms of opioid withdrawal?	14%	89%	75% (<0.001)
How familiar are you with identifying the signs and symptoms of an opioid overdose?	11%	89%	78% (<0.001)
How comfortable would you feel responding to an opioid overdose?	9%	86%	77% (<0.001)
How comfortable do you feel writing a prescription for naloxone?	11%	92%	81% (<0.001)
How comfortable do you feel typing a new prescription for naloxone?	26%	93%	67% (<0.001)

Table S2. Survey results comparing student perception post-teaching and post-assessment.

	Post-teaching answering in the affirmative	Post-assessment answering in the affirmative	Change in the proportion answering in the affirmative post-assessment and post-teaching (p-value)
How familiar are you with the risks of opioid overdose?	90%	98%	7% (<0.001)
How familiar are you with the pharmacology of naloxone?	86%	95%	10% (<0.001)
Do you believe that utilizing pharmacists to increase naloxone access can save lives?	98%	95%	-3% (0.493)
How familiar are you with the CA BOP protocol for pharmacists to furnish naloxone?	88%	94%	6% (0.004)
How comfortable do you feel talking to patients about opioids?	86%	96%	11% (<0.001)
How comfortable do you feel talking to a patient about naloxone?	86%	98%	11% (<0.001)
How comfortable do you feel counseling a patient on the administration of intramuscular naloxone in a vial with a syringe?	72%	84%	12% (<0.001)
how comfortable do you feel counseling a patient on the administration of commercially available Narcan nasal spray?	89%	98%	8% (<0.001)
How comfortable do you feel counseling a patient on the administration of naloxone nasal spray kit with atomizer/white cones?	79%	87%	7% (<0.001)
How comfortable do you feel counseling a patient on the administration of Evzio?	77%	89%	12% (<0.001)
How comfortable do you feel selecting the proper naloxone product and route for a patient?	83%	93%	10% (<0.001)
How comfortable do you feel counseling a patient on the effectiveness of naloxone?	90%	98%	7% (<0.001)
How comfortable do you feel counseling a patient on the adverse effects of naloxone?	90%	96%	6% (<0.001)
How comfortable do you feel counseling a patient on safety and tolerability of naloxone?	88%	96%	8% (<0.001)
How comfortable are you with counseling a patient on opioid overdose prevention?	87%	96%	9% (<0.001)
How familiar are you with identifying the signs and symptoms of opioid withdrawal?	89%	96%	7% (<0.001)
How familiar are you with identifying the signs and symptoms of an opioid overdose?	89%	95%	6% (<0.001)
How comfortable would you feel responding to an opioid overdose?	86%	91%	6% (<0.001)
How comfortable do you feel writing a prescription for naloxone?	92%	98%	6% (<0.001)
How comfortable do you feel typing a new prescription for naloxone?	93%	96%	4% (<0.001)

Table S3. Survey results comparing student perception pre-teaching and post-assessment.

	Pre-teaching answering in the affirmative	Post-assessment answering in the affirmative	Change in the proportion answering in the affirmative (p-value)
How familiar are you with the risks of opioid overdose?	36%	98%	61% (<0.001)
How familiar are you with the pharmacology of naloxone?	17%	95%	78% (<0.001)
Do you believe that utilizing pharmacists to increase naloxone access can save lives?	92%	95%	3% (0.005)
How familiar are you with the CA BOP protocol for pharmacists to furnish naloxone?	7%	94%	87% (<0.001)
How comfortable do you feel talking to patients about opioids?	24%	96%	72% (<0.001)
How comfortable do you feel talking to a patient about naloxone?	27%	98%	71% (<0.001)
How comfortable do you feel counseling a patient on the administration of intramuscular naloxone in a vial with a syringe?	15%	84%	69% (<0.001)
how comfortable do you feel counseling a patient on the administration of commercially available Narcan nasal spray?	23%	98%	75% (<0.001)
How comfortable do you feel counseling a patient on the administration of naloxone nasal spray kit with atomizer/white cones?	17%	87%	70% (<0.001)
How comfortable do you feel counseling a patient on the administration of Evzio?	9%	89%	80% (<0.001)
How comfortable do you feel selecting the proper naloxone product and route for a patient?	10%	93%	83% (<0.001)
How comfortable do you feel counseling a patient on the effectiveness of naloxone?	19%	98%	78% (<0.001)
How comfortable do you feel counseling a patient on the adverse effects of naloxone?	19%	96%	78% (<0.001)
How comfortable do you feel counseling a patient on safety and tolerability of naloxone?	15%	96%	81% (<0.001)
How comfortable are you with counseling a patient on opioid overdose prevention?	21%	96%	75% (<0.001)
How familiar are you with identifying the signs and symptoms of opioid withdrawal?	14%	96%	83% (<0.001)
How familiar are you with identifying the signs and symptoms of an opioid overdose?	11%	95%	84% (<0.001)
How comfortable would you feel responding to an opioid overdose?	9%	91%	83% (<0.001)
How comfortable do you feel writing a prescription for naloxone?	11%	98%	86% (<0.001)
How comfortable do you feel typing a new prescription for naloxone?	26%	96%	71% (<0.001)
